# The Impact of the SARS-Cov2 Pandemic on a Persuasive Educational Antimicrobial Stewardship Program in a University Hospital in Southern Italy: A Pre-Post Study

**DOI:** 10.3390/antibiotics10111405

**Published:** 2021-11-16

**Authors:** Margherita Macera, Lorenzo Onorato, Federica Calò, Caterina Monari, Rosa Annibale, Giuseppe Signoriello, Giovanna Donnarumma, Maria Vittoria Montemurro, Massimiliano Galdiero, Nicola Coppola

**Affiliations:** 1 Department of Mental Health and Public Medicine, Section of Infectious Diseases, University of Campania Luigi Vanvitelli, 80138 Naples, Italy; macera.margherita@libero.it (M.M.); lorenzonorato@libero.it (L.O.); fede.calo85@gmail.com (F.C.); caterina.monari@gmail.com (C.M.); 2 Pharmacy Unit, AOU Vanvitelli, 80138 Naples, Italy; rosa.annibale@policliniconapoli.it; 3 Department of Mental Health and Public Medicine-Medical Statistics Unit, University of Campania Luigi Vanvitelli, 80138 Naples, Italy; giuseppe.signoriello@unicampania.it; 4 Department of Experimental Medicine, University of Campania Luigi Vanvitelli, 80138 Naples, Italy; giovanna.donnarumma@unicampania.it (G.D.); massimiliano.galdiero@unicampania.it (M.G.); 5 Direzione Sanitaria, AOU Vanvitelli, 80138 Naples, Italy; mariavittoria.montemurro@policliniconapoli.it

**Keywords:** SARS-CoV-2, antimicrobial stewardship program, DDD, antimicrobial resistance, COVID-19

## Abstract

Objectives: We evaluated the effect of the pandemic on the disruption of a persuasive educational antimicrobial stewardship program (ASP) conducted in a university hospital in southern Italy. Methods: In March 2020, the ASP, which began in January 2017 and was carried out at different times in 10 wards, was stopped due to the COVID-19 pandemic. We conducted an observational study with interrupted time series analysis to compare the antibiotic consumption and costs, average length of hospital stay and in-hospital mortality between 12 months before and 9 months after the interruption. Results: Four medical, four surgical wards and two ICUs were included in the study, for a total of 35,921 patient days. Among the medical wards we observed after the interruption a significant increase in fluoroquinolone use, with a change in trend (CT) of 0.996, *p* = 0.027. In the surgical wards, we observed a significant increase in the overall consumption, with a change in level (CL) of 24.4, *p* = 0.005, and in the use of third and fourth generation cephalosporins (CL 4.7, *p* = 0.003). In two ICUs, we observed a significant increase in piperacillin/tazobactam and fluoroquinolone consumption (CT 9.28, *p* = 0.019, and 2.4, *p* = 0.047). In the wards with a duration of ASP less than 30 months, we observed a significant increase in antibiotic consumption in the use of piperacillin/tazobactam and fluoroquinolones (CT 12.9, *p* = 0.022: 4.12, *p* = 0.029; 1.004, *p* = 0.011). Conclusions: The interruption of ASP during COVID-19 led to an increase in the consumption of broad-spectrum antibiotics, particularly in surgical wards and in those with a duration of ASP less than 30 months.

## 1. Introduction

Antibiotic resistance is a major public health problem and antimicrobial overuse is considered the principal driver in selecting antimicrobial-resistant organisms [1]. To counter this phenomenon, antimicrobial stewardship programs (ASPs) have been launched in many hospitals. 

Since December 2019, a new beta-corona virus (SARS-CoV-2) has spread all over the world from Wuhan in China [2], causing a disease known as Coronavirus disease (COVID-19). On 30 January 2020, the World Health Organization (WHO) declared a public health emergency, and the epidemic rapidly evolved into a pandemic by March 2020 [3], with a high number of cases in the European region, especially in Italy [4]. 

It is difficult to predict the long-term impact that this pandemic may have on antimicrobial resistance (AMR). AMR can be increased by both the reduction in stewardship programs and by increasing rates of antimicrobial prescribing. For example, despite few reports of bacterial coinfection [5], almost 75% of patients with COVID-19 received antimicrobial therapy [6], and most of them were treated with broad-spectrum molecules [7]. Moreover, due to the SARS-CoV-2 pandemic, some of the ASPs were interrupted to reorganize the work activity in the hospital sector. However, there are few data on the consumption of antimicrobials during the COVID-19 pandemic, as well as in settings where ASPs were active. 

The aim of this study was to evaluate the effect of the disruption of a persuasive educational antimicrobial stewardship program conducted in a university hospital in southern Italy from January 2017 due to the SARS-CoV-2 pandemic.

## 2. Methods

From January 2017, we started a persuasive-educational ASP that took place different times in 10 wards (4r medical, 4 surgical and 2 ICUs) at the University Hospital Luigi Vanvitelli in Naples [8,9,10]. 

Briefly, we identified a multidisciplinary team, including specialists in infectious diseases, clinical microbiology, pharmacy and statistics. The intervention included periodic prospective audits conducted by the infectious disease specialists 2 to 3 times a week. During the audits, all the antibiotic prescriptions were reviewed, and the physicians in care were given recommendations on the indication, choice of antibiotics, route of administration and duration of the therapies. Although in some cases the audits were conducted with trainees, the final decision to accept the advice provided by the consultants was always taken by a senior physician. Furthermore, the infectious diseases consultants were responsible for writing and sharing diagnostic and therapeutic protocols based on national and international guidelines on the main infectious syndromes in critically ill patients, including hospital-acquired and ventilator-associated pneumonia, complicated intra-abdominal infections, catheter-related bloodstream infections, candidemias and *S. aureus* and *Enterococcus* spp. bacteremias. The clinical microbiologists provided updated reports on the susceptibility pattern of clinical isolates, while the pharmacists gave information about the antibiotic consumption of each ward and the related costs; finally, the statistician provided the data regarding hospital mortality and the length of stay. The outcomes evaluated, including antibiotic consumption, mortality rate, mean length of stay and antibiotic therapy costs, were reported to the unit involved every 4–6 months. 

Due to COVID-19 pandemic, on 28 February 2020, this program was stopped to reorganize the work activity in the hospital. Thus, in the present pre-post retrospective study, we evaluated the effect of this interruption. 

### 2.1. Outcomes

The primary outcomes evaluated were the difference in antibiotic consumption, expressed as the defined daily dose (DDD) per 100 patient days (PD) and the incidence of all bloodstream infections (BSI) (expressed as events/100 PD), as well as those caused by multidrug resistant (MDR) bacteria or Candida spp, before and after the interruption of ASP. The MDR categorization was applied according to the European Centre for Disease Control definitions [11]. The secondary outcomes included the hospital mortality rate, expressed as deaths/100 PD, the mean length of hospital stay and the antibiotic expense, expressed as euros/100 PD. All variables were collected and analysed every month. The wards were grouped according to three areas, four medical wards (nephrology, endocrinology, neurology and geriatric Units), four surgical wards (two general surgery units, thoracic surgery and gynecology and obstetrics Units) and two intensive care units. Furthermore, we analysed the data according to the duration of the stewardship program. The units were divided as follows: Group 1 (including the two ICUs, one general surgery unit, the gynecology and obstetrics and the thoracic surgery and the nephrology units), where the program had been active for more than 30 months, and Group 2 (including one general surgery unit, and endocrinology, neurology and geriatrics units), where the program was interrupted after less than 30 months of activity. The cut off of 30 months was chosen because it corresponded approximately to the median duration of ASP among the units.

The pre-interruption period (ASP-period) was defined as March 2019–February 2020; the post-interruption period (non-ASP-period) included the months from March 2020 to December 2020. In the COVID-19 (non-ASP) period, none of these wards were designated for the hospitalization of patients with a SARS-CoV-2 infection but continued to hospitalize the same type of patients as the pre-COVID-19 (ASP) period. 

### 2.2. Statistical Analysis

Continuous variables were summarized as mean and standard deviation, and categorical variables as absolute and relative frequencies. For continuous variables, the differences were evaluated by the Student’s *t*-test and categorical variables were compared by the chi-square test, using exact procedures if needed. Interrupted time series analysis was used to assess the effect of the interruption of ASP on DDDs, mortality, costs and the incidence of bloodstream infections. 

Differences were considered statistically significant at *p* < 0.05 (two-tailed tests). The analysis was performed using IBM SPSS version 21.0.

### 2.3. Ethics Approval

All methods used in the study were in accordance with the international guidelines, with the standards on human experimentation of the Ethics Committee of the Azienda Ospedaliera Universitaria, the University of Campania Luigi Vanvitelli and with the Helsinki Declaration of 1975 and revised in 1983. Since the leadership of the University of Campania formally approved and interrupted the program (statement no. 295/2017 and 5910/2020), and the data were collected during routine clinical practice, and no approval was required by the local Ethics Committee. 

## 3. Results

### 3.1. Antibiotic Consumption and Costs

Four medical wards, four surgical wards and two Intensive Care Units (ICUs) were included in the study, for a total of 35,921 patient days (PD). 

The changes in antibiotic consumption in the study periods are summarized in Table 1, Table 2 and Table 3.

With regards the medical wards (Table 1), we did not observe a significant difference in the overall antibiotic consumption between the two study periods, with a change in level (CL) from −3.1 DDD/100 PD, 95% CI −67.3 to 61.1, *p* = 0.92, and a change in trend (CT) from 15.4, CI −3.7 to 34.9, *p* = 0.11. No significant change was registered for carbapenem use (CL 3.2, 95% CI −8.9 to 15.4, *p* = 0.58), as well as for the consumption of piperacillin/tazobactam (CL −1.5, 95% CI −9.8 to 6.8, *p* = 0.7) and third and fourth generation cephalosporins (CL −3.7, 95% CI −13.9 to 6.5, *p* = 0.45). Conversely, we observed a significant increase in the trend of fluoroquinolone use (CT 0.99, 95% CI 0.13 to 1.8, *p* = 0.027) and a non-significant increase in the trend of aminopenicillin use (CT 3.2, 95% CI −0.17 to 6.67, *p* = 0.061). No variation was observed in the prescriptions of vancomycin or linezolid. 

Regarding the surgical wards (Table 2), we found a significant increase in the consumption level of all antibiotics (CL 24.4, 95% CI 8.4 to 40.4, *p* = 0.005) after the interruption of ASP, with a decrease in trend (CT −3.9, 95% CI −7.1 to −0.6, *p* = 0.022). Similarly, a significant increase in piperacillin/tazobactam prescription was observed (CT 1.12, 95% CI 0.4 to 1.8, *p* = 0.003), as well as a significant increase in the level of consumption of third and fourth generation cephalosporins (CL 4.7, 95% CI 1.9 to 7.4, *p* = 0.003) and aminopenicillins (CL 7.8, 95% CI 2.26 to 13.4, *p* = 0.009). However, a significant decrease in trend was observed for both classes, while no change was registered for carbapenem, fluoroquinolone and echinocandin use. 

Finally, in the two ICUs, we found a non-significant decrease in the level of antibiotic use from March 2020 (CL −660, 95% CI −1380 to 59.3, *p* = 0.069), with a significant increase in the consumption of piperacillin/tazobactam (CT 9.2, 95% CI 1.7 to 16.8, *p* = 0.019) and fluoroquinolones (CT 2.4, 95% CI 0.03 to 4.78, *p* = 0.047) (Table 3). No significant variation in carbapenem and cephalosporin prescriptions was observed. 

More interestingly, when we grouped the wards according to the duration of the antimicrobial stewardship program before the interruption, we observed a significant increase in the overall antibiotic consumption (CT 12.9, 95% CI 2.12 to 23.7, *p* = 0.022)among the wards where the program had been conducted for less than 30 months, as well as an increase in the use of piperacillin/tazobactam (CT 4.12, 95% CI 0.5 to 7.75, *p* = 0.029) and fluoroquinolone (CT 1.0, 95% CI 0.3 to 1.73 *p* = 0.011) (Table 4). This was not observed in the wards with a longer ASP duration (Supplementary Table S1). 

### 3.2. Bloodstream Infections 

The changes in the rate of bloodstream infections observed during the study period are summarized in Table 5.

Regarding the ICU wards, a non-significant reduction in the rate of BSI was found during the COVID pandemic (CL −13.7 events/100 PD, 95% CI −42,8 to 15.4, *p* = 0.33); similar results were observed when considering the infections due to MDR Gram-positive (CL −1.6 events/100 PD, 95% CI −6.6 to 3.3, *p* = 0.48) and Gram-negative (CL −4.9 events/100 PD, 95% CI −24.2 to 14.3, *p* = 0.58) bacteria, as well as candida (CL −3.7 events/100 PD, 95% CI −10.9 to 3.5, *p* = 0.29). 

Conversely, in the surgical units, we observed an increase in the trend of bloodstream infections (CT 0.49 events/1000 PD, 95% CI 0.06 to 0.9, *p* = 0.028), with an increase in the level of infections due to MDR organisms or candida (CT 1.4 events/100 PD, 95% CI −0.09 to 2.8, *p* = 0.063), which did not reach statistical significance. 

Finally, in medical wards, we found no significant difference in the rate of all bloodstream infections (*p* = 0.31) or in the rate of infections due to MDR organisms or candida (*p* = 0.64).

### 3.3. Mean Length of Stay, Hospital Mortality and Antibiotic Expense

No significant difference in the mean length of stay (LOS) was observed for surgical wards and ICUs, while a decrease in the trend of LOS was registered for medical wards (CT −0.7 days, 95% CI −1.2 to 0.2, *p* = 0.006) (Supplementary Table S2). A similar mortality rate was registered during the two periods in all three areas.

Finally, no significant change was observed in antibiotic costs (Table 1, Table 2 and Table 3).

## 4. Discussion

Our paper aimed at investigating the effect of the interruption of an antimicrobial stewardship program by the SARS-CoV-2 pandemic on antibiotic consumption and the rate of bloodstream infections due to multidrug resistant organisms in a teaching hospital in southern Italy. Antibiotic resistance is rising to dangerously high levels all over the world, and the SARS-CoV-2 pandemic may negatively impact this phenomenon, leading to an increase in antibiotic prescriptions, particularly in hospital settings [12]. This increase could be due both to the overuse of antimicrobials in patients with severe COVID-19 [6], as well as to the interruption of antimicrobial stewardship programs because of the redeployment of personnel to other areas. In fact, several antimicrobial stewardship activities have been stopped during the pandemic, mainly because of the reduction in available resources, as reported in a survey conducted among 95 healthcare facilities in the United Kingdom [13]. However, few data are available on the effect of this interruption. Thus, we tried to evaluate the impact of the interruption of ASP in our facility.

The present study showed that during the period of interruption, we observed an increase in the trend of fluoroquinolone use in medical wards, a significant increase in all antibiotics, and in particular of third and fourth generation cephalosporins in surgical wards and, finally, in ICUs, we observed a significant increase in the consumption of piperacillin/tazobactam and fluoroquinolone use. Conversely, a previous study conducted by our group [8] demonstrated that the implementation of an ASP in two ICUs resulted in a significant decrease in the consumption of carbapenems, fluoroquinolones and third and fourth generation cephalosporins, as well as in the rate of bloodstream infections due to MDR pathogens, and the results are in line with literature data indicating the usefulness of stewardship programs in reducing the consumption of broad-spectrum antibiotics [14,15] and the incidence of infections or colonization with antibiotic resistant organisms [16,17]. 

Most interestingly, the data from the present study suggest that the long-term effect of an ASP depended on its duration, and that only an ASP lasting for at least two years may modify the habits in the prescription of antibiotics. In fact, in the wards where the program had been active for more than 30 months, there was no increase in the overall or broad-spectrum antibiotic consumption, while in those where the program had been active for less time, the interruption was associated with an increase in the overall antibiotic consumption. In a recently published paper, Jang et al. [18] evaluated the effect of the discontinuation of a restrictive antimicrobial stewardship program on antibiotic consumption in a secondary care hospital in South Korea; after the interruption, an immediate increase in the use of restricted antibiotics both in general wards and in ICUs was observed. However, compared with the present study, Jang’s study included a shorter period of intervention (18 months versus more than 30 months) and the type of strategy used. We conducted a non-restrictive intervention based on prospective audits and feedback (PAF); although some data suggest that restrictive measures might be more effective when evaluated in the short term, this difference disappears when considering longer periods of observation. [19]. Furthermore, antimicrobial stewardship programs based on PAF have demonstrated long-term efficacy in reducing the use of targeted antimicrobials and the prevalence of resistance in clinical isolates [20]. These observations might suggest that the educational work carried out during non-restrictive interventions may achieve results over time, but that their effects are longer lasting compared to those obtained through restrictive measures. 

Finally, we found that in surgical wards, the increased use of broad-spectrum antibiotics was associated with a trend towards a higher incidence of bloodstream infections due to MDR organisms and Candida spp., which was not observed in medical wards and ICUs. However, when analyzing these results, we should take into account that in wards where the ASP had started earlier, particularly in ICUs, the ASP was associated with a greater intervention of infection control, consisting of the implementation of a hand hygiene campaign, together with increased attention to the screening and isolation of patients colonized with MDR pathogens. It is well known that better microbiological outcomes are achieved when the ASP is coupled with the implementation of infection control measures [16]. The pandemic has impacted infection control in hospital settings in different ways. Certainly, increased attention to hand hygiene and disinfection of the environment, coupled with the reduced rate of hospitalization of chronically ill patients, has contributed to limiting the spread of MDR organisms. On the other hand, the focus of healthcare workers on self-protection and the overcrowding of facilities has at times caused the occurrence of outbreaks of MDR organisms [12,21]. 

The limitations of the present study are that the time of observation after the interruption of ASP may be short, and that during the pandemic, many changes occurred at our institution, including the type of patients admitted and the organization of hospital activities, and some of them are likely to have impacted antibiotic consumption and the prevalence of resistance. Furthermore, new healthcare workers have been hired during the pandemic, and in some wards, particularly in surgical units, the number of beds and the nursing staff were reduced, and this could have impacted the prescriptive attitudes in our facilities. However, we should consider that none of the wards included in ASP were assigned to the management of COVID-19 patients, and the medical staff was not modified; thus, the decision process underlying antibiotic prescriptions is not likely to have significantly changed. 

## 5. Conclusions

Our study shows a significant increase in the consumption of broad-spectrum antibiotics in some units of our institution after the interruption of a non-restrictive stewardship program. Interestingly, this trend was not observed in wards where the program had been active for more than 30 months. More prospective studies are needed to highlight the impact of the pandemic on the ASP in the hospital setting and in the community, as well as the long-term efficacy of different stewardship strategies.

## Figures and Tables

**Table 1:** Effect of the ASP interruption during COVID-19 pandemic on antibiotic consumption in the 4 medical units included in the study.

Antibiotics	Parameter Evaluated	Effect Estimate	LCI	UCI	*p* Value
Carbapenems	Change in trend	1.69	−1.27	4.65	0.24
	Change in level	3.22	−8.98	15.41	0.58
Piperacillin/tazobactam	Change in trend	−0.45	−1.72	0.82	0.47
	Change in level	−1.52	−9.88	6.85	0.70
III/IV Generation Cephalosporins	Change in trend	1.12	−1.04	3.29	0.29
	Change in level	−3.72	−13.96	6.51	0.45
Aminopenicillins/BLI	Change in trend	3.25	−0.17	6.67	0.061
	Change in level	2.50	−14.54	19.54	0.76
Fluoroquinolones	Change in trend	0.99	0.13	1.86	0.027
	Change in level	−0.94	−5.2	3.28	0.64
Vancomycin	Change in trend	0.49	−0.68	1.68	0.38
	Change in level	−1.93	−7.29	3.43	0.46
Linezolid	Change in trend	0.43	−0.58	1.43	0.38
	Change in level	1.29	1.29	2.38	0.59
Macrolides	Change in trend	0.069	−0.24	0.38	0.64
	Change in level	−0.011	−0.29	0.27	0.94
All antibiotics	Change in trend	15.44	−3.75	34.6	0.11
	Change in level	−3.08	−67.29	61.12	0.92
Costs	Change in trend	180.3	−108.2	468.8	0.20
	Change in level	−95.5	−1535.8	1344.9	0.89

**Table 2:** Effect of the ASP interruption during COVID-19 pandemic on antibiotic consumption in the 4 surgical units included in the study.

Antibiotics	Parameter Evaluated	Effect Estimate	LCI	UCI	*p* Value
Carbapenems	Change in trend	0.20	−0.29	0.69	0.41
	Change in level	1.59	−0.65	3.84	0.15
Piperacillin/tazobactam	Change in trend	1.12	0.44	1.80	0.003
	Change in level	−1.19	−4.70	2.32	0.48
III/IV generation cephalosporins	Change in trend	−0.89	−1.43	−0.36	0.003
	Change in level	4.69	1.88	7.49	0.003
Aminopenicillins/BLI	Change in trend	−1.36	−2.43	−0.30	0.016
	Change in level	7.86	2.27	13.46	0.009
Fluoroquinolones	Change in trend	−0.18	−0.48	0.13	0.23
	Change in level	1.17	−0.43	2.76	0.14
Vancomycin	Change in trend	−0.06	−0.32	0.21	0.64
	Change in level	0.29	−1.08	1.66	0.66
Linezolid	Change in trend	−0.07	−0.25	0.10	0.4
	Change in level	0.91	−0.02	1.83	0.054
Tigecyclin	Change in trend	0.06	−0.17	0.29	0.58
	Change in level	0.57	−0.62	1.75	0.32
Cefazolin	Change in trend	−2.21	−5.07	0.65	0.12
	Change in level	3.78	−10.46	18.03	0.58
Metronidazole	Change in trend	−0.50	−0.92	−0.07	0.024
	Change in level	3.0	0.76	5.24	0.012
Echinocandins	Change in trend	−0.06	−0.44	0.32	0.74
	Change in level	1.36	−0.31	3.04	0.10
All antibiotics	Change in trend	−3.89	−7.15	−0.63	0.022
	Change in level	24.4	8.42	40.43	0.005
Costs	Change in trend	134	−143.1	411.2	0.32
	Change in level	276.7	621.9	1175	0.52

**Table 3:** Effect of the ASP interruption during COVID-19 pandemic on antibiotic consumption in the 2 ICUs included in the study.

Antibiotics	Parameter Evaluated	Effect Estimate	LCI	UCI	*p* Value
Carbapenems	Change in trend	−2.7	−11.3	5.93	0.5
	Change in level	−23.81	−70.17	22.5	0.3
Piperacillin/tazobactam	Change in trend	9.3	1.75	16.8	0.019
	Change in level	8.2	−31.4	47.8	0.66
III/IV generation Cephalosporins	Change in trend	−11.2	−43.7	21.3	0.47
	Change in level	−156.0	−328.1	16.0	0.07
Aminopenicillins/BLI	Change in trend	8.8	−23.4	41.1	0.57
	Change in level	−139.4	−309.9	31.1	0.10
CEF/BLI	Change in trend	−2.6	25.8	2.7	0.81
	Change in level	−49.4	−157.9	59.0	0.35
Fluoroquinolones	Change in trend	2.4	0.03	4.7	0.047
	Change in level	−10.7	−23.1	1.7	0.08
Tigecyclin	Change in trend	0.13	−3.1	3.4	0.93
	Change in level	−11.1	−27.9	5.8	0.18
Vancomycin	Change in trend	−4.2	−14.9	6.4	0.41
	Change in level	−46.1	−102.6	10.3	0.10
Linezolid	Change in trend	1.33	−1.8	4.5	0.38
	Change in level	−1.99	−18.5	14.5	0.8
Daptomycin	Change in trend	−2.3	−5.0	0.45	0.09
	Change in level	17.4	2.9	31.7	0.021
Colistin	Change in trend	−7.9	−25.6	9.6	0.35
	Change in level	−67.3	−158.9	24.3	0.14
Macrolides	Change in trend	5.4	1.1	9.7	0.018
	Change in level	−6.5	−28.5	15.3	0.53
Echinocandins	Change in trend	−0.9	−10.2	8.5	0.85
	Change in level	−43.3	−92.9	6.3	0.08
All antibiotics	Change in trend	10.2	−125.3	145.6	0.87
	Change in level	−660.5	−1380.3	59.3	0.07
Costs	Change in trend	−200.1	−5330	5730	0.94
	Change in level	−14,605	−4275.9	13,765.6	0.29

**Table 4:** Effect of the ASP interruption during COVID-19 pandemic on antibiotic consumption in the 4 units where the program had been active for less than 30 months.

Antibiotics	Parameter Evaluated	Effect Estimate	LCI	UCI	*p* Value
Carbapenems	Change in trend	1.51	−1.30	4.32	0.27
	Change in level	5.30	−5.63	16.2	0.32
Piperacillin/tazobactam	Change in trend	4.11	0.48	7.75	0.029
	Change in level	−3.13	−13.5	7.30	0.53
III/IV Generation cephalosporins	Change in trend	0.67	−0.57	1.91	0.27
	Change in level	−2.08	−8.47	4.31	0.50
Aminopenicillins/BLI	Change in trend	1.65	−0.15	3.46	0.07
	Change in level	8.39	−1.18	17.9	0.08
Fluoroquinolones	Change in trend	1.004	0.27	1.74	0.011
	Change in level	−0.95	−4.61	2.71	0.59
Vancomycin	Change in trend	0.53	−0.61	1.68	0.34
	Change in level	−1.09	−6.21	4.04	0.66
Linezolid	Change in trend	0.23	−0.55	1.00	0.54
	Change in level	2.14	−1.87	6.16	0.27
Cefazolin	Change in trend	0.17	−1.61	1.96	0.84
	Change in level	−3.69	−13.02	5.64	0.41
All antibiotics	Change in trend	12.9	2.12	23.7	0.022
	Change in level	−0.16	−47.6	47.3	0.99
Costs	Change in trend	358.4	50.94	665.9	0.026
	Change in level	640.3	−419.7	1700.4	0.22

**Table 5:** Effect of the ASP interruption during COVID-19 pandemic on the rate of bloodstream infections (BSI) in medical, surgical and critical areas.

		Parameter Evaluated	Effect Estimate	LCI	UCI	*p* Value
Medical wards	All BSI	Change in trend	0.03	−0.02	0.08	0.25
		Change in level	−0.13	−0.39	0.13	0.31
	MDRO BSI and candidemia	Change in trend	0.004	−0.02	0.03	0.77
		Change in level	−0.03	−0.18	0.11	0.64
Surgical wards	All BSI	Change in trend	0.049	0.006	0.09	0.028
		Change in level	0.21	−0.01	0.45	0.059
	MDRO BSI and candidemia	Change in trend	0.008	−0.02	0.04	0.53
		Change in level	0.138	−0.009	0.24	0.06
ICUs	All BSI	Change in trend	−0.15	−5.7	5.4	0.95
		Change in level	−13.7	−42.8	15.4	0.33
	MDR Gram-negative BSI	Change in trend	−0.11	−3.87	3.66	0.95
		Change in level	−4.94	−24.2	14.3	0.59
	MDR Gram-positive BSI	Change in trend	−0.33	−1.29	0.63	0.47
		Change in level	−1.65	−6.58	3.27	0.48
	Candidemia	Change in trend	−0.012	−1.4	1.38	0.98
		Change in level	−3.72	−10.97	3.54	0.29

## Data Availability

The data presented in this study are available on request from the corresponding author.

## Supplementary Materials

The following are available online at https://www.mdpi.com/article/10.3390/antibiotics10111405/s1, Table S1: Effect of the ASP interruption during COVID-19 pandemic on antibiotic consumption in the 6 units where the program had been active for more than 30 months, Table S2: Effect of the ASP interruption during COVID-19 pandemic on the length of hospital stay and mortality in medical, surgical and critical areas.
