# Supplementary material for: The Impact of the SARS-Cov2 Pandemic on a Persuasive Educational Antimicrobial Stewardship Program in a University Hospital in Southern Italy: A Pre-Post Study
Source: Antibiotics (Basel). 2021 Nov 16;10(11):1405. doi: 10.3390/antibiotics10111405 (PMC8614883; doi:10.3390/antibiotics10111405)
Supplement: Supplementary file 1 [file antibiotics-10-01405-s001.zip › antibiotics-1429782-SI.pdf]

Table S1. Effect of the ASP interruption during COVID-19 pandemic on antibiotic consumption in the 6 units where the program had been active for more than 30 months.

Antibiotics	Parameter Evaluated	Effect Estimate	LCI	UCI	*p* Value
**Carbapenems**	Change in trend	-0.40	-1.23	0.42	0.31
	Change in level	2.48	-1.83	6.78	0.21
**Piperacillin/tazobactam**	Change in trend	1.16	-0.22	2.54	0.09
	Change in level	3.62	-3.38	10.62	0.29
**III/IV Generation Cephalosporins**	Change in trend	-2.13	-3.31	-0.94	**0.002**
	Change in level	-3.49	-9.57	2.60	0.24
**Aminopenicillins/BLI**	Change in trend	-0.48	-1.49	0.53	0.32
	Change in level	4.25	-1.06	9.57	0.11
**Fluoroquinolones**	Change in trend	-0.09	-0.56	0.37	0.67
	Change in level	0.58	-1.73	2.88	0.60
**Tigecyclin**	Change in trend	0.04	-0.22	0.29	0.75
	Change in level	0.39	-0.91	1.70	0.53
**Vancomycin**	Change in trend	-0.52	-1.08	0.04	0.07
	Change in level	-0.74	-3.61	2.13	0.59
**Linezolid**	Change in trend	0.046	-0.45	0.54	0.84
	Change in level	1.74	-0.80	4.28	0.16
**Daptomycin**	Change in trend	-0.58	-1.18	0.02	0.57
	Change in level	2.91	-0.48	6.30	0.09
**Macrolides**	Change in trend	0.002	-0.58	0.59	0.99
	Change in level	-0.17	-3.04	2.69	0.90
**Echinocandins**	Change in trend	-0.28	-0.77	0.21	0.24
	Change in level	0.38	-2.97	2.21	0.76
**All antibiotics**	Change in trend	-6.01	-13.34	1.33	0.10
	Change in level	7.36	-29.7	44.4	0.68
**Costs**	Change in trend	-46.95	-313.6	219.7	0.71
	Change in level	-402.2	-1480.4	975.9	0.54

Table S2. Effect of the ASP interruption during COVID-19 pandemic on the length of hospital stay and mortality in medical, surgical and critical areas.

	Parameter Evaluated	Effect Estimate	LCI	UCI	*p* Value
**LOS (medical wards)**	Change in trend	-0.74	-1.23	-0.24	0.006
	Change in level	1.23	-1.39	3.87	0.33
**LOS (surgical wards)**	Change in trend	-0.03	-0.12	0.14	0.71
	Change in level	-0.36	-1.3	0.57	0.42
**LOS (ICUs)**	Change in trend	-2.27	-4.83	0.29	0.078
	Change in level	5.9	-7.5	19.3	0.36
**Mortality (ICUs)**	Change in trend	0.75	-0.1	1.61	0.08
	Change in level	-1.54	-5.75	2.66	0.44
